# Edge-Aware Dynamic Convolution and Cross-Modal Relation-Aware Fusion Network for Infrared and Visible Image Fusion

**DOI:** 10.3390/s26196166

**Published:** 2026-09-29

**Authors:** Shunli Liu, Anjie Chen, Qiao Luo, Zhengyong Feng, Weichao Yang

**Affiliations:** School of Electronic Information Engineering, China West Normal University, Nanchong 637009, China; liusl16@stu.cwnu.edu.cn (S.L.); chenanjie@stu.cwnu.edu.cn (A.C.); cwnuluoqiao@stu.cwnu.edu.cn (Q.L.)

**Keywords:** deep learning, infrared and visible image fusion, edge-aware dynamic convolution, cross-modal relation-aware, complementarity consistency loss

## Abstract

Infrared and visible image fusion combines complementary thermal and structural information from the two modalities into a single composite image. Existing methods have two critical limitations: (1) inadequate utilization of visible structural information causes blurred edges, and (2) modality-specific and shared responses are not always separately represented when learning the fusion weights. To address these problems, an edge-aware dynamic convolution and cross-modal relation-aware fusion network is proposed to fully exploit visible structural details and explicitly model complementary information. Specifically, an edge-aware dynamic convolution is designed to extract edge features from visible images for generating dynamic convolution kernels, which adaptively enhance edge details during fusion. Then, a cross-modal relation-aware adaptive fusion module is designed to explicitly compute difference and interaction features of infrared and visible features and predicts adaptive fusion weights to balance modality-specific information. In addition, a complementarity consistency loss is introduced to jointly constrain intensity, edge and complementary information. Experiments on several public datasets demonstrate that the proposed method provides better visual quality and quantitative performance.

## 1. Introduction

Infrared and visible image fusion combines complementary information from two imaging modalities to form a unified scene representation [1]. Infrared images provide stable thermal responses and can reveal targets under weak or uneven illumination [2]. Visible images, in contrast, offer richer textures, sharper contours, and more complete scene structures [3]. An effective fusion result should therefore retain the thermal information of the infrared modality while preserving the spatial details of the visible modality. Owing to this complementary property, infrared and visible image fusion has been applied to object detection, visual surveillance, autonomous driving, and scene understanding [4].

Conventional infrared and visible image fusion approaches often separate the input images into several components and merge them according to predefined manners. Common methods are based on multi-scale transforms, sparse representation, saliency analysis, or optimization models [5]. Among them, multi-scale transform methods process low-frequency intensity components and high-frequency structural components with different fusion manners [6]. Sparse representation methods describe image patches with dictionaries and fuse their sparse coefficients [7]. Saliency-based methods assign larger weights to thermal targets or prominent structures, while optimization-based methods introduce regularization terms to preserve intensity, edges, and local structures [8]. However, their reliance on hand-crafted features and fixed fusion manners limits their adaptability to complex scenes and may lead to information loss, low contrast, or visual artifacts. Learning-based spatial–frequency representations have also been explored in remote sensing image analysis to improve feature modeling across different image resolutions [9].

Recent studies have increasingly applied deep learning to infrared and visible image fusion [10,11]. These methods usually extract multimodal features, fuse them, and reconstruct the final image. Feature-guided reconstruction has also been explored in related low-level vision tasks. Dong et al. introduced a feature-masking layer into a denoising convolutional neural network to suppress noise in portable ultrasound images while retaining important feature information [12]. In infrared and visible image fusion, however, visible structural information is often used mainly as an input feature or auxiliary attention map rather than to adapt reconstruction to local structures. Because edges and textures vary across image regions, applying the same convolution kernel at every spatial location may weaken object boundaries and fine details. Dynamic filter networks and kernel prediction networks address this limitation by generating input-dependent or spatially varying filters [13,14]. These studies motivate the use of visible edge information to guide spatially adaptive reconstruction in infrared and visible image fusion.

Beyond local reconstruction, fusion quality also depends on how information from the two modalities is balanced. Attention-based methods such as DATFuse and MMAE learn dependencies between infrared and visible features and use them to guide fusion [11,15]. Infrared and visible features contain modality-specific responses, including thermal targets and visible textures, together with structures shared by the two modalities [16]. We therefore consider both the difference and interaction between the two feature streams when estimating the fusion weights.

**Figure 1:**
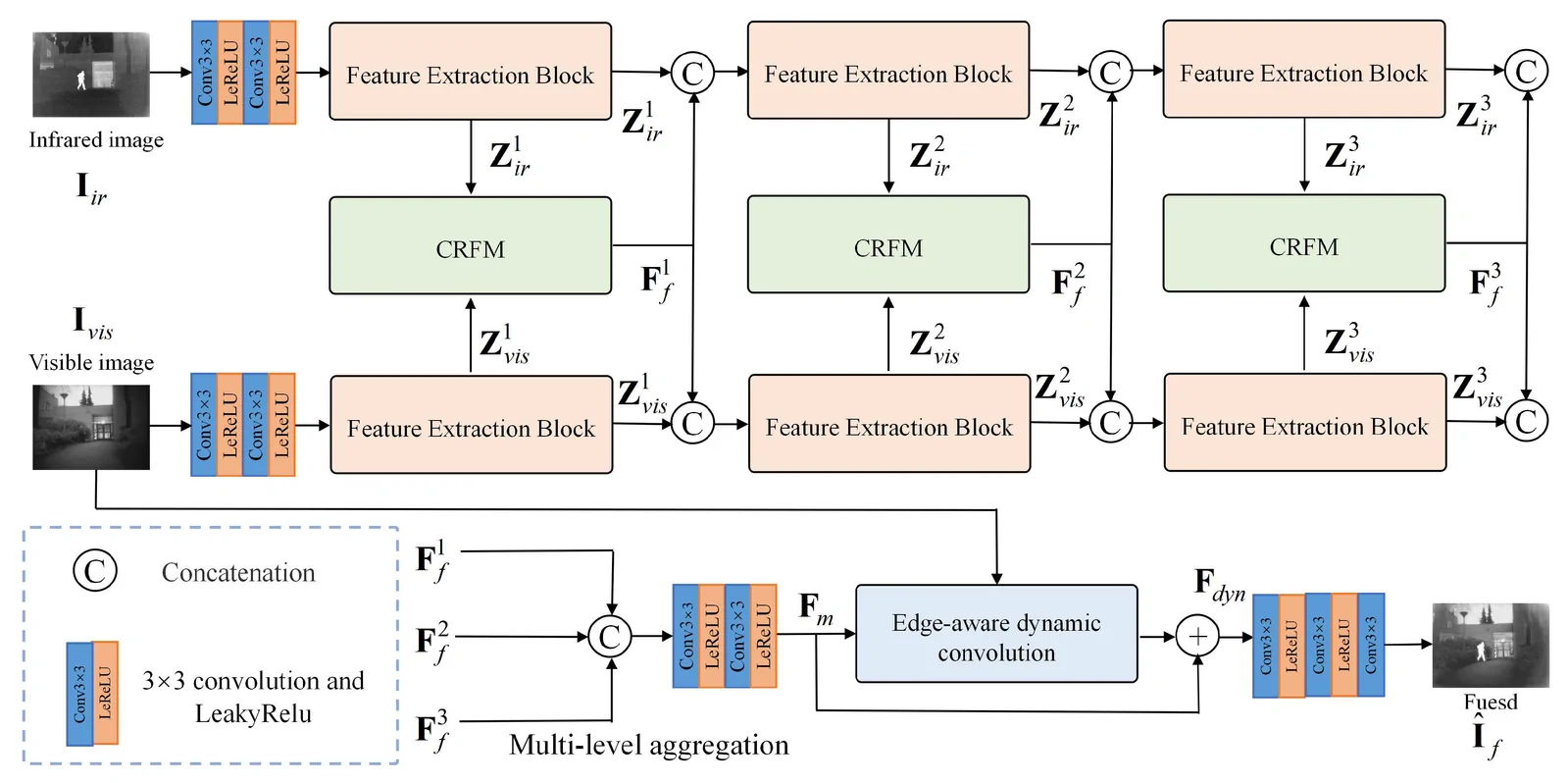
Overall architecture of the proposed edge-aware dynamic convolution and cross-modal relation-aware fusion network. Infrared and visible images are first processed by two modality-specific branches. Cross-modal relation-aware fusion modules (CRFMs) are used at different stages to select complementary and shared information. The multi-scale fused features are then reconstructed by the edge-aware dynamic convolution module to generate the final fused image.

To address these problems, we propose an edge-aware dynamic convolution and cross-modal relation-aware fusion network. As shown in Figure 1, the infrared and visible images are processed by two modality-specific branches through three successive feature-extraction and fusion stages. At each stage, a cross-modal relation-aware fusion module estimates the modality weights from the difference and interaction between the two feature streams, and the fused feature is fed back to both branches before the next stage. The three stage-level fusion features are then aligned and aggregated. The aggregated feature and visible edge map are used to predict a separate 3×3 convolution kernel at each spatial position, after which a three-layer reconstruction head produces the final fused image. Training is guided by a complementarity consistency loss composed of intensity, edge, and thermal target terms. The main contributions are listed below.

- An edge-aware dynamic reconstruction module is developed for infrared and visible image fusion. It uses visible edge features to generate pixel-wise kernels, allowing the reconstruction operation to adapt to local boundaries and textures.
- A cross-modal relation-aware fusion mechanism is introduced to derive the modality weights jointly from the feature difference and element-wise interaction. Its stage-wise feedback design returns the selected fusion information to both modality branches before subsequent feature extraction.
- A complementarity consistency objective combines a positive-detail intensity target, signed gradient constraints, and a target-masked thermal penalty. It preserves useful infrared responses and visible structures while limiting the transfer of unnecessary infrared intensity to background regions.

## 2. Materials and Methods

### 2.1. Dataset Description

Three public datasets are employed in the experiments, including M3FD, RoadScene, and TNO. M3FD is used for model training, validation, and same-dataset testing. RoadScene and TNO are used only for cross-dataset testing. This setting evaluates both the fusion performance and the generalization ability.

The M3FD dataset [17] contains aligned infrared and visible images collected under different illumination, weather, and scene conditions. In this work, 3900 image pairs from the M3FD Detection subset are used. They are divided into 3500 training pairs and 400 validation pairs. The object bounding-box annotations provided with M3FD Detection are used during training to construct the object-region weighting map for the thermal target loss. A separate M3FD Fusion subset containing 100 infrared-visible image pairs is used as the test dataset. All images in M3FD Fusion are excluded from the training and validation sets to avoid data overlap.

The RoadScene dataset [18] contains aligned infrared and visible images of road environments, including vehicles, pedestrians, buildings, and other urban structures. Forty image pairs are selected from RoadScene to evaluate the model on data not used during training or model selection.

The TNO dataset [19] provides aligned thermal and visible image pairs from various surveillance and nighttime environments. The scenes contain different targets, backgrounds, and lighting conditions. In this study, 40 image pairs from TNO are used to evaluate the model without additional training or fine-tuning on this dataset.

### 2.2. Data Processing Methods

The infrared and visible images are processed as paired inputs throughout training and testing. During training, four corresponding patches with a spatial size of 256×256 pixels are cropped from each of the 3500 training image pairs. The same spatial position is used for the infrared and visible images to maintain their pixel-level correspondence. Therefore, a total of 14,000 paired training patches are obtained. The four patches are sampled in a cyclic order from target regions, visible-detail regions, infrared-detail regions, and randomly selected regions. During training, the paired patches are randomly flipped horizontally with a probability of 0.5. Gamma and contrast adjustments are independently applied to the infrared and visible patches, each with a probability of 0.5. The gamma values are sampled from [0.85,1.15], and the contrast factors from [0.90,1.10]. The remaining 400 pairs from M3FD Detection are used as the validation set to detect model performance and select the trained model. The M3FD Fusion, RoadScene, and TNO test images are not used for network optimization or validation. This separation prevents information leakage between the training and test sets. Random cropping is applied only during network training. During testing, no random cropping is performed. Each complete infrared-visible image pair is directly fed into the network, and the fused image is reconstructed at the original spatial size. The 100 M3FD Fusion pairs are used to evaluate the fusion performance on the main dataset. The 40 RoadScene pairs and 40 TNO pairs are used to evaluate how the model performs on unseen datasets without additional training. The visible image is converted from RGB to YCbCr space. The infrared image and the luminance channel of the visible image are fed into the network. After fusion, the chrominance channels of the visible image are combined with the fused luminance channel to reconstruct the final RGB image. All loss terms and objective metrics are calculated on the luminance or grayscale image.

## 3. Methodology

### 3.1. Overall Architecture

Given a registered infrared image $I_{ir}\in R^{1\times H\times W}$ and a visible image $I_{vis}\in R^{1\times H\times W}$, the proposed network generates a fused image ${\hat{I}}_{f}\in R^{1\times H\times W}$. Here, *H* and *W* denote the image height and width, respectively. As shown in Figure 1, the network contains two shallow feature extractors, three modality feature extraction stages, three cross-modal relation-aware fusion modules, a multi-level aggregation module, an edge-aware dynamic convolution module, and a reconstruction head.

The infrared and visible images are first mapped into the feature space:

(1) $$F_{ir}^{0}=S_{ir}\left(I_{ir}\right),\;F_{vis}^{0}=S_{vis}\left(I_{vis}\right),$$

where $S_{ir}\left(\cdot \right)$ and $S_{vis}\left(\cdot \right)$ are independently learned shallow feature-extraction operators for the infrared and visible branches, respectively. Each operator is implemented by two consecutive 3×3 convolutional layers. $F_{ir}^{0},F_{vis}^{0}\in R^{C\times H\times W}$ are the initial modality features. Here, *C* is a fixed architectural setting that specifies the number of output channels of the first 3×3 convolution and, consequently, the channel dimension of the initial features. It is set to 24 in this work. For example, an input of size 1×256×256 is mapped to a feature tensor of size 24×256×256. Separate extractors are adopted to handle the different features of infrared and visible images.

The two modality branches are then processed through three successive stages. At the *l*-th stage, the infrared and visible features are first updated by their corresponding feature extraction blocks:

(2) $$Z_{m}^{l}=E_{m}^{l}\left(F_{m}^{l-1}\right),\;m\in \left\{ir,vis\right\},\;l\in \left\{1,2,3\right\},$$

where $E_{m}^{l}\left(\cdot \right)$ denotes the feature extraction block of modality *m* at stage *l*. $Z_{ir}^{l}$ and $Z_{vis}^{l}$ are the intermediate infrared and visible features produced at this stage.

The relationship between the two modalities is then modeled by the cross-modal relation-aware fusion module:

(3) $$F_{f}^{l}=R^{l}\left(Z_{ir}^{l},Z_{vis}^{l}\right),$$

where $R^{l}\left(\cdot \right)$ denotes the cross-modal relation-aware fusion module at stage *l*. $F_{f}^{l}$ is the fused feature obtained from the two modality streams. The relation cues computed within the module guide the adaptive weighting of these features.

The fusion feature is further fed back to both modality branches:

(4) $$F_{m}^{l}=Z_{m}^{l}+G_{m}^{l}\left(\left[Z_{m}^{l},F_{f}^{l}\right]\right),\;m\in \left\{ir,vis\right\},$$

where [·] denotes channel-wise concatenation and $G_{m}^{l}\left(\cdot \right)$ denotes the feedback projection block. The projected fusion information is added to the original modality feature through a residual connection. In this way, the infrared branch can receive useful visible structures, while the visible branch can receive useful infrared responses. The updated features $F_{ir}^{l}$ and $F_{vis}^{l}$ are then passed to the next stage.

The three fusion features describe multimodal information at different representation levels. They are aligned and aggregated as

(5) $$F_{ms}=A\left(\left[U_{1}\left(F_{f}^{1}\right),U_{2}\left(F_{f}^{2}\right),U_{3}\left(F_{f}^{3}\right)\right]\right),$$

where $U_{l}\left(\cdot \right)$ denotes the alignment projection associated with the fusion feature from stage *l*. The three projections have the same role and mapping form, and the subscript identifies the stage-wise feature being processed. Because the stage features in the proposed architecture are spatially aligned, this mapping is implemented mainly as a channel projection. A(·) denotes the multi-level aggregation module, and $F_{ms}$ is the aggregated fusion feature.

To guide local reconstruction, structural edges are extracted from the visible image and used together with the aggregated feature:

(6) $$E_{vis}=G_{e}\left(I_{vis}\right),\;F_{dyn}=D\left(F_{ms},E_{vis}\right),$$

where $G_{e}\left(\cdot \right)$ denotes the visible edge extractor, $E_{vis}\in R^{1\times H\times W}$ is the single-channel visible edge feature, and D(·) denotes the edge-aware dynamic convolution module. This module predicts location-dependent convolution kernels from the current fusion feature and visible edge information. The resulting feature $F_{dyn}\in R^{C\times H\times W}$ therefore contains enhanced local boundaries and textures.

Finally, the aggregated feature and the dynamically reconstructed feature are combined to generate the fused image:

(7) $${\hat{I}}_{f}=O\left(F_{ms}+F_{dyn}\right),$$

where O(·) denotes the reconstruction head, which comprises three consecutive 3×3 convolutional layers. The first two layers are followed by LeakyReLU activations, and the final layer produces the single-channel fused image. The residual addition preserves the main scene content from $F_{ms}$, while $F_{dyn}$ provides additional edge and texture details. The final fused image therefore maintains infrared thermal saliency and visible structural information.

### 3.2. Cross-Modal Relation-Aware Fusion Module

**Figure 2:**
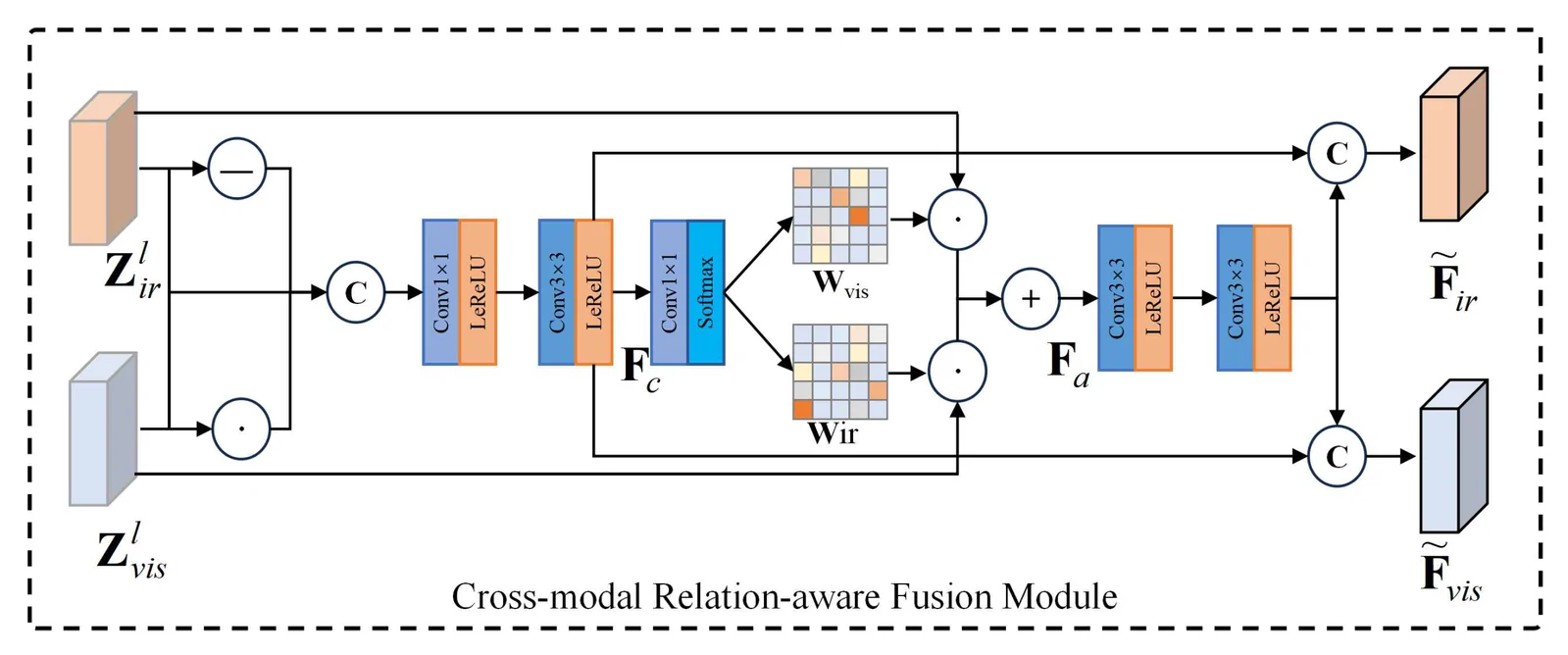
Structure of the proposed cross-modal relation-aware fusion module. The difference and interaction features are computed to provide relative discrepancy and element-wise interaction cues, respectively. These cues are used to predict spatial and channel fusion weights. The weighted features are fused and fed back to the two modality branches for further feature extraction.

Infrared and visible features contain both different and shared information. Infrared features mainly describe thermal responses, while visible features provide textures and structural details. To model these relationships, a cross-modal relation-aware adaptive fusion module is designed, as shown in Figure 2. Attention-based fusion methods such as DATFuse [11] and MMAE [15] learn dependencies between the two modalities to guide feature integration. Our module follows this cross-modal perspective by making the relationship explicit through a signed difference and an element-wise interaction between the two feature maps. These two cues are jointly encoded to generate an adaptive weight map for each modality, and the resulting fused feature is fed back to both branches before the next stage.

Given the visible feature $Z_{vis}^{l}\in R^{C\times H\times W}$ and the infrared feature $Z_{ir}^{l}\in R^{C\times H\times W}$, the difference feature and interaction feature are calculated as

(8) $$F_{d}=Z_{vis}^{l}-Z_{ir}^{l},\;F_{i}=Z_{vis}^{l}⊙Z_{ir}^{l},$$

where ⊙ denotes element-wise multiplication. The difference feature $F_{d}$ captures the signed discrepancy between the visible and infrared features, whereas the interaction feature $F_{i}$ captures their element-wise interaction. These two relation cues are jointly used to estimate the fusion weights.

The two relation features are concatenated and encoded by convolutional layers:

(9) $$F_{c}=\phi_{r}\left(\left[F_{d},F_{i}\right]\right),$$

where [·] denotes channel-wise concatenation. The relation encoder $\phi_{r}\left(\cdot \right)$ consists of a 1×1 convolution, a LeakyReLU activation, a 3×3 convolution, and another LeakyReLU activation. The 1×1 convolution combines the two relation features, while the 3×3 convolution further captures their local spatial relationships. $F_{c}$ is the encoded cross-modal relation feature.

The relation feature is then mapped into two adaptive weight maps:

(10) $$\left[W_{vis},W_{ir}\right]={\mathrm{Softmax}}_{m}\left(\phi_{w}\left(F_{c}\right)\right),$$

where $\phi_{w}\left(\cdot \right)$ denotes a 1×1 convolution and ${\mathrm{Softmax}}_{m}\left(\cdot \right)$ is applied along the modality dimension. $W_{vis}$ and $W_{ir}$ are the adaptive weights for the visible and infrared features, respectively. They have the same spatial size as the input features and satisfy

(11) $$W_{vis}+W_{ir}=1.$$

Thus, the contributions of the two modalities can be adjusted according to the current feature responses.

The visible and infrared features are weighted separately and then added:

(12) $$F_{a}=W_{vis}⊙Z_{vis}^{l}+W_{ir}⊙Z_{ir}^{l},$$

where $F_{a}$ denotes the adaptively weighted feature. The weighted feature is further refined as

(13) $$F_{f}=\phi_{f}\left(F_{a}\right),$$

where $\phi_{f}\left(\cdot \right)$ contains two consecutive 3×3 convolution and LeakyReLU layers. $F_{f}$ is the relation-aware fused feature. It contains selected infrared thermal information, visible structural details, and shared scene information.

As shown in Figure 2, the fused feature is further fed back to both modality branches. It is concatenated with the original visible and infrared features, respectively:

(14) $$\begin{array}{cc} {\tilde{F}}_{vis}\; & =\phi_{vis}\left(\left[Z_{vis}^{l},F_{f}\right]\right),\\ {\tilde{F}}_{ir}\; & =\phi_{ir}\left(\left[Z_{ir}^{l},F_{f}\right]\right), \end{array}$$

where $\phi_{vis}\left(\cdot \right)$ and $\phi_{ir}\left(\cdot \right)$ are modality-specific refinement blocks. Each block contains two 3×3 convolution and LeakyReLU layers. ${\tilde{F}}_{vis}$ and ${\tilde{F}}_{ir}$ are the refined visible and infrared features, respectively. The fused information is therefore used to improve both branches before the next feature extraction stage.

### 3.3. Edge-Aware Dynamic Convolution

**Figure 3:**
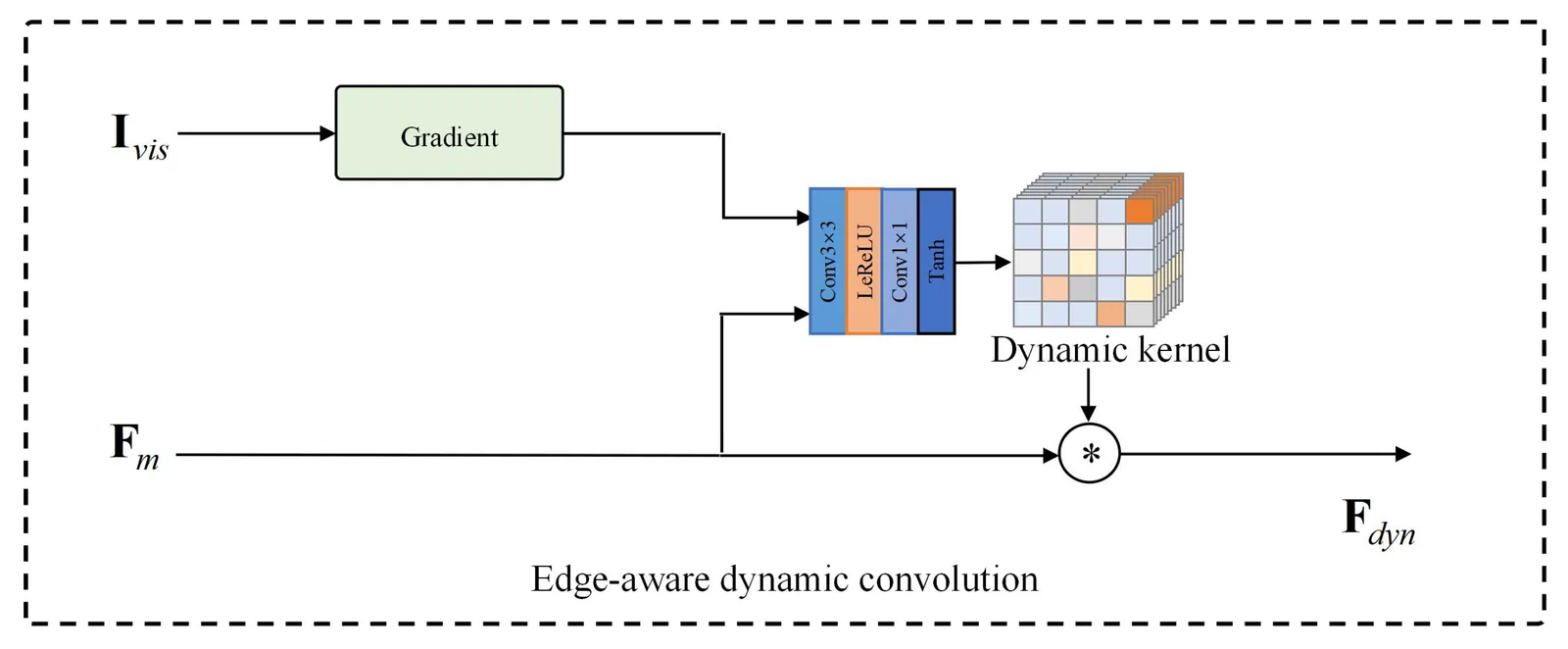
Structure of the proposed edge-aware dynamic convolution. Visible gradients are combined with the multi-level fusion feature to generate a pixel-wise dynamic kernel for each spatial position. The generated kernels are then applied to local neighborhoods of the fusion feature to obtain the edge-enhanced output. The symbol ∗ denotes the dynamic convolution operation.

Fixed convolution kernels apply the same operation to all image regions and may weaken local boundaries and textures. To address this problem, an edge-aware dynamic convolution is designed as shown in Figure 3. It takes the visible image $I_{vis}$ and the multi-level fusion feature $F_{ms}$ as inputs. Visible gradients are used to guide the generation of pixel-wise dynamic kernels, which are then applied to $F_{ms}$.

The horizontal and vertical gradients of the visible image are first extracted as

(15) $$G_{x}=S_{x}*I_{vis},\;G_{y}=S_{y}*I_{vis},$$

where $S_{x}$ and $S_{y}$ denote the horizontal and vertical gradient operators, respectively, and ∗ denotes spatial convolution.

The visible edge map is then calculated by

(16) $$E_{vis}=\sqrt{G_{x}^{2}+G_{y}^{2}+\varepsilon },$$

where $E_{vis}\in R^{1\times H\times W}$ represents the visible edge response, and ε is a small positive constant used to maintain numerical stability in locally flat regions. In this work, ε is set to $10^{-6}$.

The edge map is combined with the fusion feature to predict a dynamic kernel for each spatial position:

(17) $$K=\mathrm{Reshape}\left(\phi_{k}\left(\left[F_{ms},E_{vis}\right]\right)\right)\in R^{H\times W\times k^{2}},$$

where [·] denotes channel-wise concatenation and $\phi_{k}\left(\cdot \right)$ denotes the kernel prediction network. K contains one k×k dynamic kernel for each spatial position. In this work, *k* is set to 3.

Finally, the predicted kernels are applied to the local neighborhoods of $F_{ms}$:

(18) $$F_{dyn}\left(c,p\right)=\sum_{q\in \Omega }K_{p,q}\;F_{ms}\left(c,p+q\right),$$

where *p* denotes the current spatial position, *c* denotes the channel index, and Ω denotes the k×k neighborhood around *p*. $K_{p,q}$ is the kernel weight predicted for position *p* and neighborhood offset *q*. The output $F_{dyn}$ therefore uses different convolution kernels in different regions, which helps preserve visible boundaries and fine textures during fusion.

### 3.4. Complementarity Consistency Loss

To guide end-to-end training, a complementarity consistency loss is introduced. This loss jointly considers intensity preservation, edge consistency, and complementary information. It consists of an intensity loss, an edge loss, and a thermal target loss.

A positive-detail target is first constructed as

(19) $$T^{+}=I_{vis}+\mathrm{ReLU}\left(I_{ir}-I_{vis}\right),$$

where $I_{ir}$ and $I_{vis}$ denote the infrared and visible images, respectively. The ReLU(·) function keeps only positive infrared responses. Thus, $T^{+}$ preserves the visible image as the basic structure and adds useful thermal information where the infrared intensity is higher.

The intensity loss is defined as

(20) $$L_{int}=\frac{1}{N}{∥{\hat{I}}_{f}-T^{+}∥}_{1},$$

where ${\hat{I}}_{f}$ is the fused image, N=H×W is the number of pixels, and ${∥\cdot ∥}_{1}$ denotes the $L_{1}$ norm. This term preserves the basic intensity, visible details, and useful infrared responses.

The edge loss constrains the horizontal and vertical gradients:

(21) $$L_{edge}=\frac{1}{N}\left({∥\nabla_{x}{\hat{I}}_{f}-\nabla_{x}T^{+}∥}_{1}+{∥\nabla_{y}{\hat{I}}_{f}-\nabla_{y}T^{+}∥}_{1}\right),$$

where $\nabla_{x}$ and $\nabla_{y}$ denote the signed horizontal and vertical gradient operators, respectively. This term preserves edge strength and direction and reduces edge reversal in the fused image.

A thermal target loss is further introduced as

(22) $$\begin{array}{c} M_{h}=M_{t}⊙I\left(I_{ir}>I_{vis}\right),\\ L_{th}=\frac{1}{N}{∥M_{h}⊙\mathrm{ReLU}\left(I_{ir}-{\hat{I}}_{f}\right)∥}_{1}, \end{array}$$

where $M_{t}\in {\left[0,1\right]}^{H\times W}$ is obtained from the object bounding boxes in the M3FD Detection subset. The bounding boxes are converted into a binary mask, smoothed using a 15×15 Gaussian kernel, and normalized. I(·) and ⊙ denote the element-wise indicator function and element-wise multiplication, respectively. The resulting $M_{h}$ weights target regions where the infrared intensity is higher than the visible intensity and is used only to calculate $L_{th}$ during training.

The total loss is written as

(23) $$L_{total}=L_{int}+L_{edge}+L_{th},$$

The three loss terms jointly preserve image intensity, structural edges, and infrared thermal targets.

## 4. Results

### 4.1. Experimental Setup

The network is developed in PyTorch 2.6.0 and trained on an NVIDIA RTX 4090 GPU. The network uses 24 feature channels in each modality branch and is trained with a batch size of 16. Adam [20] is used for optimization with $\beta_{1}=0.9$, $\beta_{2}=0.999$, $\epsilon =10^{-8}$, and zero weight decay. The learning rate is initialized at $5\times 10^{-6}$, and a cosine annealing strategy [21] reduces it to $1\times 10^{-6}$ over 100 training epochs. The infrared and visible images are provided as paired inputs. Two separate shallow feature extractors are used to map them into the feature space. Three cross-modal relation-aware fusion modules are employed at different stages to progressively model the difference and interaction between the two modalities. In the edge-aware dynamic convolution module, Sobel operators are used to extract visible gradients, and a pixel-wise 3×3 dynamic kernel is generated for each spatial position.

### 4.2. Comparison Methods and Metrics

Eight representative methods are selected for comparison: DenseFuse [22], U2Fusion [23], YDTR [24], EMMA [25], CAF [26], LUT-Fuse [27], GIFNet [28], and DAFusion [29]. DenseFuse adopts a densely connected encoder–decoder structure. U2Fusion provides an unsupervised framework for several image fusion tasks. YDTR introduces a Y-shaped dynamic Transformer to model local and contextual features. EMMA applies equivariant self-supervised learning to multimodal fusion. CAF jointly performs image fusion and downstream perception tasks. LUT-Fuse transfers fusion knowledge into learnable look-up tables for fast inference. GIFNet exploits the relations among low-level vision tasks, while DAFusion uses domain adaptation to improve performance under changing imaging conditions.

Six metrics are used for quantitative evaluation: standard deviation (SD), mutual information (MI) [30], visual information fidelity (VIF) [31], $Q^{AB/F}$ [32], normalized mutual information (NMI) [33], and gradient-based feature mutual information (${\mathrm{FMI}}_{G}$) [34]. SD reflects image contrast and intensity variation. MI and NMI measure the information retained from the source images. VIF evaluates visual information preservation. $Q^{AB/F}$ describes the transfer of source edges, while ${\mathrm{FMI}}_{G}$ measures the preservation of gradient features and local structures.

For all comparison methods, we used the official implementations and pretrained models released by the original authors, together with their recommended inference settings. Each method was tested on the same registered infrared and visible image pairs, and the resulting images were evaluated at the original resolution using the same metric code.

### 4.3. Comparison on the M3FD Dataset

#### 4.3.1. Quantitative Comparison on the M3FD Dataset

Table 1 presents the quantitative comparison results on the M3FD dataset. The best and second-best results are highlighted in bold and underlined, respectively. The proposed method achieves the best results on five of the six evaluation metrics. Specifically, it obtains an MI of 4.7724, a VIF of 0.7526, a $Q^{AB/F}$ of 0.6373, an NMI of 0.6884, and an ${\mathrm{FMI}}_{G}$ of 2.6535. These results show that the proposed method can preserve more source information while maintaining good visual quality, edge information, and structural details. Compared with LUT-Fuse, which obtains the second-best MI and NMI values, the proposed method improves MI from 4.1720 to 4.7724 and NMI from 0.6027 to 0.6884. This indicates that more information from the infrared and visible images is transferred to the fused image. The improvement mainly benefits from the cross-modal relation-aware fusion module. The difference feature provides a signed discrepancy between the two feature streams, whereas the interaction feature records their element-wise relationship. These two cues are jointly used to predict adaptive fusion weights, helping the network balance thermal responses and visible structures. The proposed method also achieves a $Q^{AB/F}$ value of 0.6373, which is higher than the second-best value of 0.6140 obtained by EMMA. This result shows that the source edges are more effectively transferred to the fused image. In addition, the proposed method improves ${\mathrm{FMI}}_{G}$ from 2.0592 to 2.6535 compared with EMMA. The improvement in gradient-based feature mutual information demonstrates that more local edges and texture structures are preserved. This result verifies the effectiveness of the edge-aware dynamic convolution. For the VIF metric, the proposed method achieves the highest value of 0.7526. This means that the fused image contains more visually useful information and has better overall visual fidelity. The SD value of the proposed method is 39.0047, which is lower than the value of 42.3750 obtained by EMMA but higher than most other comparison methods. SD mainly reflects the gray-level variation and contrast of an image. A very high SD does not always mean that the fusion result is visually better, because excessive contrast may also enlarge noise or cause unnatural intensity changes. The proposed method achieves a competitive SD while obtaining the best results on the other five metrics. This demonstrates that it provides a better balance between image contrast, thermal saliency, information preservation, and structural fidelity.

#### 4.3.2. Qualitative Comparison on the M3FD Dataset

**Figure 4:**
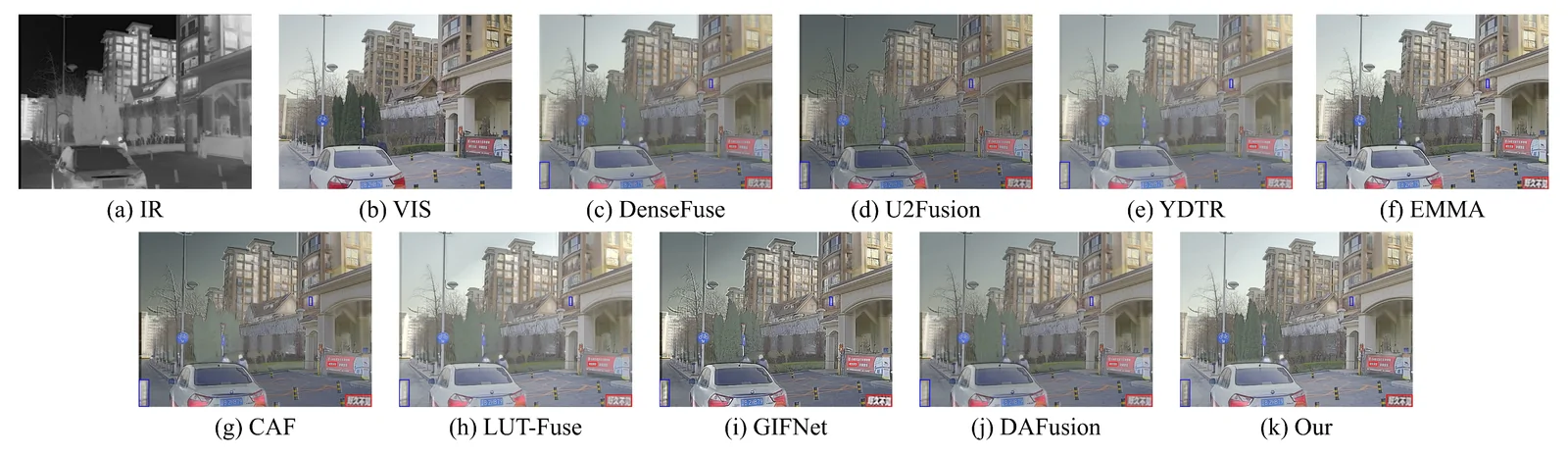
Qualitative comparison of different infrared and visible image fusion methods for a street scene from the M3FD dataset. (**a**) Infrared image, (**b**) visible image, and fusion results generated by (**c**) DenseFuse, (**d**) U2Fusion, (**e**) YDTR, (**f**) EMMA, (**g**) CAF, (**h**) LUT-Fuse, (**i**) GIFNet, (**j**) DAFusion, and (**k**) Our.

**Figure 5:**
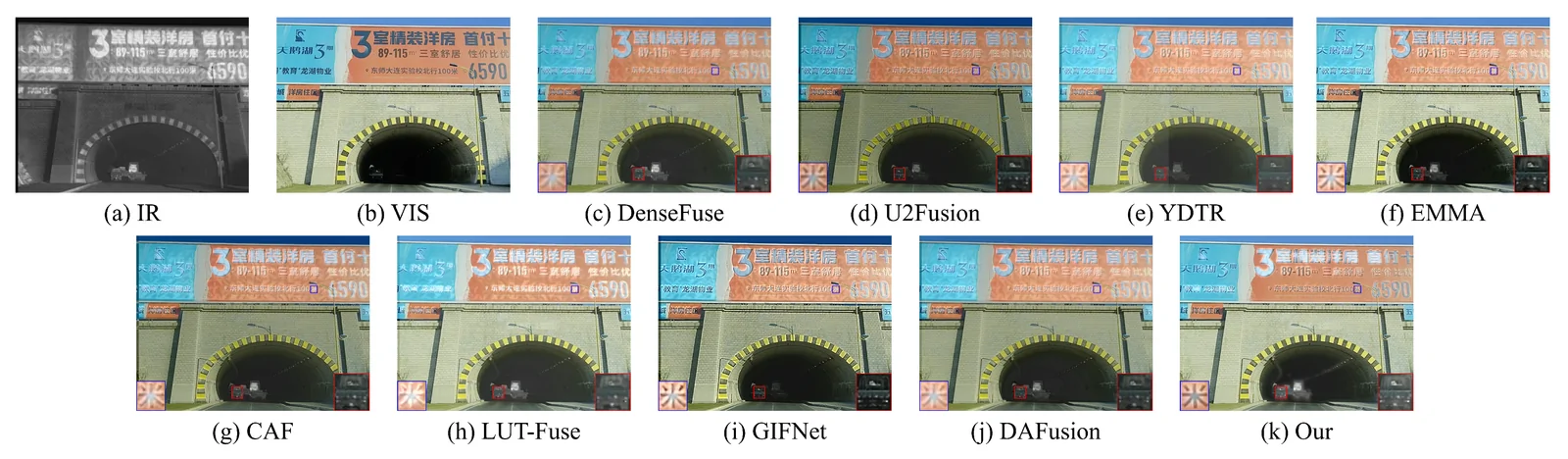
Qualitative comparison of different infrared and visible image fusion methods for a tunnel scene from the M3FD dataset. (**a**) Infrared image, (**b**) visible image, and fusion results generated by (**c**) DenseFuse, (**d**) U2Fusion, (**e**) YDTR, (**f**) EMMA, (**g**) CAF, (**h**) LUT-Fuse, (**i**) GIFNet, (**j**) DAFusion, and (**k**) Our.

The qualitative comparison results on the M3FD dataset are shown in Figure 4 and Figure 5. The infrared images provide clear thermal responses, while the visible images contain richer textures and scene structures. A good fusion method should preserve both types of information without introducing obvious artifacts. As shown in the full-image results, some comparison methods cannot achieve a good balance between the two modalities. DenseFuse preserves the main scene content, but some local boundaries and fine textures are relatively smooth. U2Fusion retains visible structures in several regions, but the responses of some thermal targets are weakened. YDTR and CAF provide acceptable overall fusion results, but local details are not always clear. LUT-Fuse produces strong intensity responses, while some visible textures are not fully preserved. GIFNet may generate large gray-level variations, but some local structures appear less natural. DAFusion preserves useful information from both modalities, but slight detail loss can still be observed in complex regions. In contrast, the proposed method produces clearer object boundaries and more complete texture details. The thermal responses remain visible, while the background structures are not covered by unnecessary infrared information. In the enlarged regions of Figure 4 and Figure 5, the proposed method preserves sharper edges and more continuous local structures. Fine visible details are retained without weakening salient infrared targets. These visual improvements are mainly produced by the cooperation of the two proposed modules. The cross-modal relation-aware fusion module selects useful information according to the relationship between the two modalities. It strengthens modality-specific information and removes part of the redundant response. The edge-aware dynamic convolution further uses visible edge features to control local reconstruction. Different image regions are processed by different dynamic kernels. Therefore, flat regions remain stable, while edges and texture-rich regions receive stronger structure-aware reconstruction. The qualitative results are consistent with the improvements in VIF, $Q^{AB/F}$, and ${\mathrm{FMI}}_{G}$.

### 4.4. Generalization Comparison on the TNO and RoadScene Datasets

#### 4.4.1. Quantitative Comparison on the TNO and RoadScene Datasets

To examine how the model performs on data not used during training, the model trained on M3FD was directly evaluated on 40 image pairs from TNO and 40 image pairs from RoadScene. No additional training or fine-tuning was performed on either dataset, and neither dataset was used during network optimization or model selection. The quantitative results are reported in Table 2 and Table 3. Each value represents the average result over the 40 test pairs from the corresponding dataset.

On the TNO dataset, the proposed method obtains the highest MI, VIF, $Q^{AB/F}$, NMI, and ${\mathrm{FMI}}_{G}$ values, reaching 3.4628, 0.7198, 0.5124, 0.5142, and 1.8467, respectively. EMMA obtains the highest SD value of 49.3862, whereas the proposed method achieves an SD of 42.0835. These results show that the proposed method retains more information and structural detail than the comparison methods on the 40 TNO test pairs.

On the RoadScene dataset, the proposed method achieves the highest MI, VIF, NMI, and ${\mathrm{FMI}}_{G}$ values, with scores of 3.8427, 0.6835, 0.5481, and 1.7086, respectively. U2Fusion obtains the highest $Q^{AB/F}$ value of 0.4938, while EMMA obtains the highest SD value of 55.9863. The proposed method achieves an SD of 41.4862 and maintains a good balance between information preservation, visual fidelity, and structural detail on the 40 RoadScene test pairs.

#### 4.4.2. Qualitative Comparison on the TNO and RoadScene Datasets

**Figure 6:**
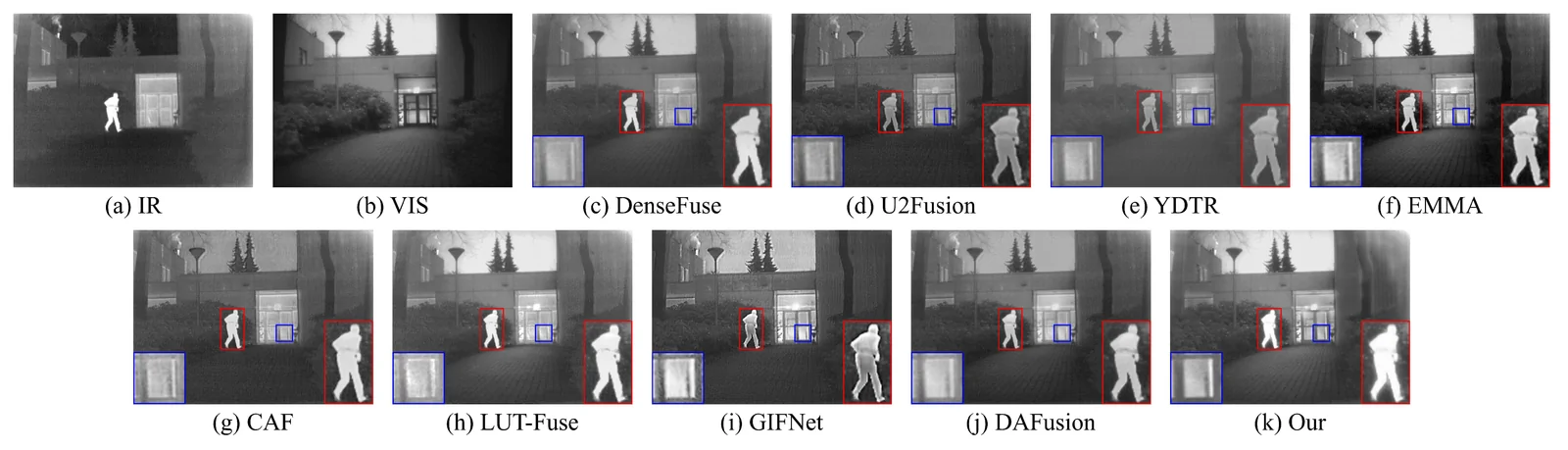
Qualitative generalization comparison of different infrared and visible image fusion methods on the TNO dataset. (**a**) Infrared image, (**b**) visible image, and fusion results generated by (**c**) DenseFuse, (**d**) U2Fusion, (e) YDTR, (**f**) EMMA, (**g**) CAF, (**h**) LUT-Fuse, (**i**) GIFNet, (**j**) DAFusion, and (**k**) Our.

**Figure 7:**
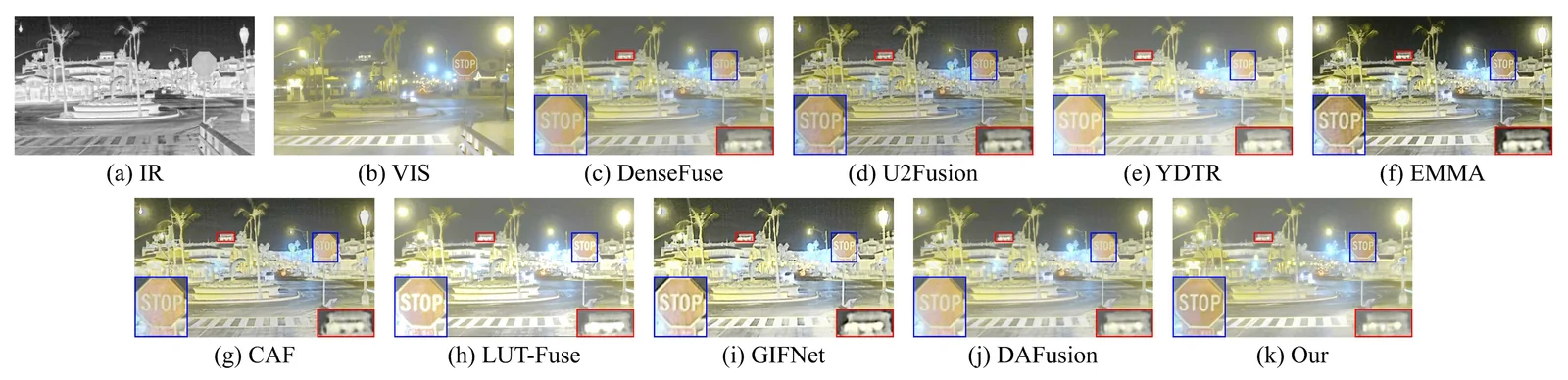
Qualitative generalization comparison of different infrared and visible image fusion methods on the RoadScene dataset. (**a**) Infrared image, (**b**) visible image, and fusion results generated by (**c**) DenseFuse, (**d**) U2Fusion, (**e**) YDTR, (**f**) EMMA, (**g**) CAF, (**h**) LUT-Fuse, (**i**) GIFNet, (**j**) DAFusion, and (**k**) Our.

The qualitative results on the TNO and RoadScene datasets are shown in Figure 6 and Figure 7, respectively. Differences in the visual results directly reflect their cross-dataset generalization ability.

For the TNO dataset, some comparison methods preserve the main thermal responses but lose visible textures in the background. Other methods retain more visible structures but weaken the contrast of thermal regions. Several methods also produce smooth boundaries or uneven intensity distributions. These problems indicate that fixed fusion rules learned from the training dataset may not adapt well to unseen image conditions. The proposed method preserves clear thermal responses while maintaining the main visible structures. The edges in the enlarged regions are sharper and more continuous. Background textures are also retained with less smoothing. At the same time, strong infrared responses are mainly kept in useful target regions, and unnecessary infrared intensity is not widely introduced into the background. These observations agree with the best $Q^{AB/F}$ and ${\mathrm{FMI}}_{G}$ values obtained on TNO. 

For the RoadScene dataset, the source images contain complex road structures, objects, and background regions with different intensity distributions. Some comparison methods generate strong overall contrast but lose weak local textures. Some methods preserve visible details but provide insufficient thermal saliency. In addition, local artifacts or blurred structures can be observed in several fused results. The proposed method produces a more balanced visual result. Important infrared responses remain clear, while road boundaries, object contours, and background structures are preserved. The enlarged regions show that the proposed method maintains more complete local details and avoids obvious over-smoothing. Although its SD and $Q^{AB/F}$ values are not the highest on RoadScene, its visual result contains more complete information from the two modalities. This is consistent with its best MI, VIF, NMI, and ${\mathrm{FMI}}_{G}$ values.

Overall, the results in Figure 6 and Figure 7 show that the proposed method remains effective when the test data come from unseen datasets. The network can adaptively select complementary multimodal information and reconstruct local structures according to the current scene. These results further demonstrate that the proposed method has good robustness and cross-dataset generalization ability.

### 4.5. Robustness to Input Misregistration

The sensitivity to input alignment was evaluated on the M3FD Fusion test set. The model trained on M3FD was tested with relative offsets of 0, 2, 4, and 8 pixels between the paired infrared and visible inputs. The corresponding average results are listed in Table 4. MI, VIF, $Q^{AB/F}$, NMI, and ${\mathrm{FMI}}_{G}$ decrease gradually as the offset increases, whereas SD changes only slightly. In particular, MI decreases from 4.7724 at 0 pixels to 4.7416 at 8 pixels, while VIF decreases from 0.7526 to 0.7391 and $Q^{AB/F}$ decreases from 0.6373 to 0.6239. These results indicate that small misregistration has a limited influence on the overall fusion quality, whereas larger offsets affect the preservation of fine cross-modal structures.

### 4.6. Robustness Under Challenging Visible Conditions

To examine the effect of visible-image degradation, four degraded versions of each visible image in the M3FD Fusion test set were prepared while keeping the infrared image, image registration, model weights, and testing procedure unchanged. The degradation was applied to the visible RGB image before conversion to YCbCr. Low illumination was simulated using a gamma transformation with γ=1.8. Blur was introduced using a 7×7 Gaussian filter with σ=2.0. Rain was generated by overlaying bright slanted streaks with an angle of $-20^{\circ }$, a line width of 1 pixel, a length of 15 pixels, a target coverage of 0.0008, and an opacity of 0.25. Fog was generated using an atmospheric-scattering model with atmospheric light A=255 and transmission t=0.75. A fixed random seed of 2026 was used when generating the rain patterns. The degraded luminance channel was used as the visible input, and the corresponding chrominance channels were retained for RGB reconstruction.

The results are reported in Table 5. Compared with the original condition, low illumination and rain produce clearer decreases in MI, NMI, and VIF. Blur has a stronger effect on $Q^{AB/F}$ and ${\mathrm{FMI}}_{G}$, reflecting the loss of local edge information. Fog causes a smaller decrease in MI and NMI but still reduces SD and VIF. These results show that the proposed method can still produce fused images under the tested visible-image degradations, although the benefit of visible-gradient guidance becomes weaker when visible structures are seriously blurred, darkened, or obscured.

### 4.7. Computational Complexity Comparison

The computational cost of each method was measured on the M3FD Fusion test set using the same registered infrared–visible image pairs at their original resolution. The results are summarized in Table 6, including the parameter count, FLOPs, peak GPU memory, and inference speed.

The proposed method contains 0.5189M parameters and requires 463.672G FLOPs. Its peak GPU memory consumption is 1331.3 MiB, and its inference speed is 14.5 FPS. The pixel-wise dynamic kernels introduce additional computational cost compared with lightweight methods such as DenseFuse and LUT-Fuse. However, the proposed method remains less demanding in parameter count and FLOPs than several heavier methods, including U2Fusion, GIFNet, and DAFusion.

## 5. Discussion

### 5.1. Ablation Study of CRFM and EDC

**Figure 8:**
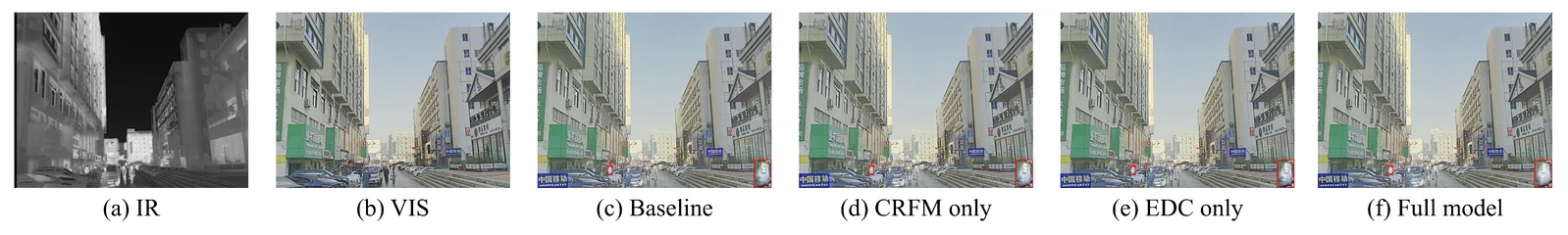
Qualitative ablation results of the proposed network modules on the M3FD dataset. (**a**) Infrared image, (**b**) visible image, (**c**) baseline without CRFM and EDC, (**d**) model with only CRFM, (**e**) model with only EDC, and (**f**) full model with both modules.

Table 7 evaluates the contributions of CRFM and EDC. In the baseline, the fused feature at each CRFM stage is obtained by equal-weight averaging of the infrared and visible branch features. The subsequent operations in CRFM are replaced by conventional convolutional layers with the same parameter budget. The EDC module is replaced by a conventional fixed-kernel convolutional residual connection with a matched parameter count. The two modality stems, multiscale merging layers, reconstruction head, loss function, and training settings remain unchanged. The baseline without either module obtains an MI of 4.4225, a VIF of 0.6739, a $Q^{AB/F}$ of 0.6065, and an ${\mathrm{FMI}}_{G}$ of 2.2416. Adding CMRAFB improves all six metrics, showing that modeling the difference and interaction between infrared and visible features supports more effective information selection. Adding EDC also improves all metrics, confirming that visible gradients and pixel-wise dynamic kernels help preserve local edges and textures. The full model achieves the best results on all metrics. Compared with the baseline, MI increases from 4.4225 to 4.7724, VIF from 0.6739 to 0.7526, $Q^{AB/F}$ from 0.6065 to 0.6373, and ${\mathrm{FMI}}_{G}$ from 2.2416 to 2.6535. These results indicate that the two modules are complementary. CRFM selects useful multimodal information, while EDC improves its local structural reconstruction. The visual results in Figure 8 are consistent with the quantitative results. The baseline contains relatively weak boundaries and textures. CRFM improves the balance between thermal responses and visible information, while EDC produces clearer local structures. The full model provides the most complete textures, clear boundaries, and natural background appearance, demonstrating the effectiveness of combining relation-aware fusion with dynamic reconstruction.

### 5.2. Ablation Study of the Loss Function

**Figure 9:**
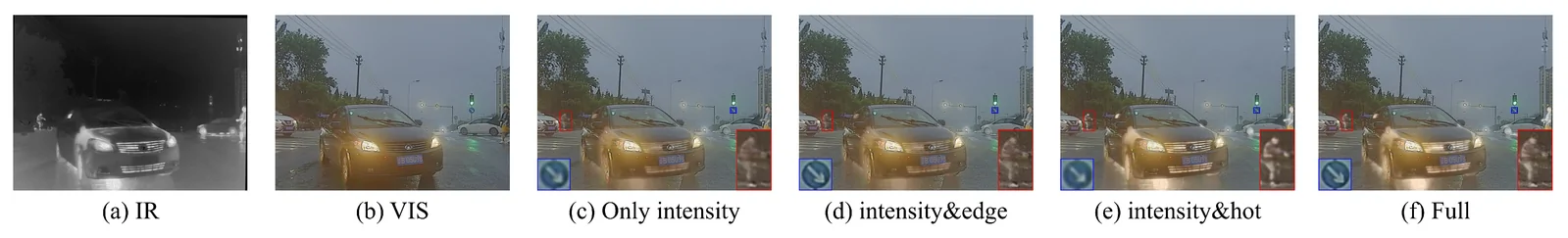
Qualitative ablation results of different loss terms on the M3FD dataset. (**a**) Infrared image, (**b**) visible image, (**c**) model trained with only $L_{int}$, (**d**) model trained with $L_{int}+L_{edge}$, (**e**) model trained with $L_{int}+L_{th}$, and (**f**) full model trained with $L_{int}+L_{edge}+L_{th}$.

Table 8 evaluates the contribution of each loss term. Using only $L_{int}$ gives relatively low VIF, $Q^{AB/F}$, and ${\mathrm{FMI}}_{G}$ values, showing that intensity supervision alone is insufficient for preserving edges and local details. After $L_{edge}$ is added, VIF increases from 0.4583 to 0.7314, $Q^{AB/F}$ from 0.2379 to 0.6371, and ${\mathrm{FMI}}_{G}$ from 1.1160 to 2.5214. This confirms that signed gradient constraints effectively preserve edge strength and direction. Adding $L_{th}$ to $L_{int}$ improves MI, SD, and NMI, indicating that more useful infrared thermal information is retained. The complete loss achieves the best results on all six metrics. Compared with $L_{int}+L_{edge}$, the full loss further improves MI from 4.5001 to 4.7724, NMI from 0.6512 to 0.6884, and ${\mathrm{FMI}}_{G}$ from 2.5214 to 2.6535. These results show that the three loss terms provide complementary supervision for intensity, structure, and thermal targets. The visual results in Figure 9 support the quantitative results. The intensity-only result contains weak boundaries and blurred local details. Adding the edge loss produces clearer contours, while adding the thermal loss strengthens the target response. The complete loss provides the best balance between thermal saliency, clear boundaries, and visible structural details.

### 5.3. Ablation of Internal Module Designs

We examined the internal choices of CRFM and EDC on the M3FD Fusion test set. Except for the component being evaluated, the network structure and training protocol were kept unchanged.

Table 9 compares three configurations of the relation representation in CRFM. Using only the difference feature or only the interaction feature leads to lower values than using both features. The combination of the two features gives the highest scores on all six metrics, showing that they provide complementary cues for estimating the fusion weights.

We also varied the number of fusion stages from one to five, as shown in Table 10. The three-stage configuration achieves the best overall results, while adding a fourth or fifth stage does not lead to further improvement.

The influence of the dynamic-kernel size is presented in Table 11. Among the tested sizes, the 3×3 kernel gives the highest MI, VIF, $Q^{AB/F}$, NMI, and ${\mathrm{FMI}}_{G}$ values. Larger kernels increase SD but reduce the other information- and structure-related metrics. We therefore retain the 3×3 kernel in the final model.

### 5.4. Limitations and Future Work

Despite its favorable performance, the current study still has several constraints. The model is trained primarily on M3FD, while its cross-dataset performance is examined only on TNO and RoadScene. Further tests on data collected by different sensors and under varied weather, lighting, and scene conditions are required. Moreover, the current framework relies on well-aligned infrared and visible image pairs. Misregistration may produce inaccurate cross-modal relation features and inconsistent edge guidance. Future work will therefore consider joint registration and fusion for unregistered or slightly misaligned image pairs.

The edge-aware dynamic convolution mainly relies on visible gradients, which may become unreliable under very low illumination, blur, rain, or fog. Pixel-wise dynamic kernels also increase computation and memory costs for high-resolution images. Moreover, the thermal target loss may provide weak guidance when thermal targets have low contrast or when the background contains strong infrared responses. Its effectiveness also depends on the object-region weighting map derived from the annotations. Gaussian smoothing softens the box boundaries, but missed targets or incorrectly included regions can still reduce the reliability of this supervision. Future work will explore more robust multimodal edge guidance, lightweight dynamic kernel prediction, and adaptive thermal region estimation. The current evaluation focuses on image-level fusion quality and does not directly measure the effect of the fused images on downstream tasks. Future work will combine the proposed fusion method with task-specific models, such as object detectors and semantic segmentation networks, to further examine the practical value of the preserved infrared and visible information.

## 6. Conclusions

In this paper, an edge-aware dynamic convolution and cross-modal relation-aware fusion network is proposed for infrared and visible image fusion. The edge-aware dynamic convolution uses visible gradients to generate pixel-wise dynamic kernels, which improves the preservation of object boundaries and local details. The cross-modal relation-aware fusion module explicitly models the difference and interaction between infrared and visible features, allowing the contributions of the two modalities to be adaptively balanced. A complementarity consistency loss is further introduced to jointly constrain image intensity, edge structure, and thermal responses. Experimental results on public datasets demonstrate that the proposed method effectively preserves infrared thermal targets and visible structural details, while achieving competitive visual quality and quantitative performance.

## Figures and Tables

**Table 1:** Quantitative comparison on the M3FD dataset. Bold indicates the best result, while underline indicates the second-best result. Metrics marked with ↑ are higher-is-better metrics.

Method	MI ↑	SD ↑	VIF ↑	$Q^{\mathrm{AB}/F}↑$	NMI ↑	${\mathrm{FMI}}_{G}↑$
DenseFuse	3.6273	31.9390	0.6323	0.4642	0.5314	1.7333
U2Fusion	2.4513	27.0413	0.5981	0.5613	0.3640	1.6567
YDTR	3.1464	31.7145	0.5934	0.4484	0.4606	1.5974
EMMA	4.0466	**42.3750**	0.7463	0.6140	0.5762	2.0592
CAF	2.6511	34.2429	0.5712	0.5269	0.3839	1.5179
LUT-Fuse	4.1720	37.4526	0.6354	0.4923	0.6027	1.5034
GIFNet	2.4645	38.2090	0.5504	0.4927	0.3556	1.4052
DAFusion	3.0302	31.4848	0.6341	0.5392	0.4433	1.7909
Ours	**4.7724**	39.0047	**0.7526**	**0.6373**	**0.6884**	**2.6535**

**Table 2:** Quantitative generalization comparison on the TNO dataset based on 40 test image pairs. Bold indicates the best result, while underline indicates the second-best result. Metrics marked with ↑ are higher-is-better metrics.

Method	MI ↑	SD ↑	VIF ↑	$Q^{\mathrm{AB}/F}↑$	NMI ↑	${\mathrm{FMI}}_{G}↑$
DenseFuse	2.9614	32.6918	0.6468	0.4217	0.4459	1.1968
U2Fusion	1.7436	24.2085	0.5191	0.3989	0.2678	0.9057
YDTR	2.2941	28.0462	0.5684	0.3578	0.3491	1.0716
EMMA	2.9197	**49.3862**	0.7098	0.4408	0.4189	1.2649
CAF	2.1664	32.9425	0.5379	0.4143	0.3298	0.8795
LUT-Fuse	3.4498	40.7161	0.6404	0.4469	0.5057	0.9976
GIFNet	2.0039	41.1738	0.4771	0.3440	0.2897	0.7084
DAFusion	2.3157	33.6912	0.6107	0.4428	0.3445	1.0631
Ours	**3.4628**	42.0835	**0.7198**	**0.5124**	**0.5142**	**1.8467**

**Table 3:** Quantitative generalization comparison on the RoadScene dataset based on 40 test image pairs. Bold indicates the best result, while underline indicates the second-best result. Metrics marked with ↑ are higher-is-better metrics.

Method	MI ↑	SD ↑	VIF ↑	$Q^{\mathrm{AB}/F}↑$	NMI ↑	${\mathrm{FMI}}_{G}↑$
DenseFuse	3.3518	36.5041	0.6409	0.4187	0.4722	1.4825
U2Fusion	2.8467	35.3824	0.5871	**0.4938**	0.4011	1.4426
YDTR	3.0214	36.8642	0.5946	0.4089	0.4258	1.3819
EMMA	3.2947	**55.9863**	0.6748	0.4396	0.4485	1.3108
CAF	3.1176	42.9315	0.6041	0.4817	0.4312	1.3744
LUT-Fuse	3.7925	44.9827	0.6164	0.4228	0.5401	1.1376
GIFNet	2.8241	50.6849	0.5260	0.3612	0.3890	1.0529
DAFusion	3.0486	42.5038	0.6291	0.4543	0.4241	1.4127
Ours	**3.8427**	41.4862	**0.6835**	0.4246	**0.5481**	**1.7086**

**Table 4:** Robustness of the proposed method to controlled input misregistration on the M3FD Fusion test set. Bold indicates the best result. Metrics marked with ↑ are higher-is-better metrics.

Displacement	MI ↑	SD ↑	VIF ↑	$Q^{\mathrm{AB}/F}↑$	NMI ↑	${\mathrm{FMI}}_{G}↑$
0 pixels	**4.7724**	**39.0047**	**0.7526**	**0.6373**	**0.6884**	**2.6535**
2 pixels	4.7684	38.4420	0.7471	0.6322	0.6862	2.6524
4 pixels	4.7589	38.4602	0.7435	0.6331	0.6856	2.6479
8 pixels	4.7416	38.5097	0.7391	0.6239	0.6843	2.6326

**Table 5:** Robustness of the proposed method under challenging visible-image conditions on the M3FD Fusion test set. Bold indicates the best result. Metrics marked with ↑ are higher-is-better metrics.

Condition	MI ↑	SD ↑	VIF ↑	$Q^{\mathrm{AB}/F}↑$	NMI ↑	${\mathrm{FMI}}_{G}↑$
Original	**4.7724**	**39.0047**	**0.7526**	**0.6373**	**0.6884**	**2.6535**
Blur	4.6542	37.2936	0.7428	0.4349	0.6847	1.8376
Fog	4.7131	30.8783	0.7299	0.5861	0.6537	2.3964
Low illumination	3.6068	38.3879	0.6547	0.5945	0.5221	2.0294
Rain	3.7214	32.9184	0.7003	0.5701	0.5619	2.1069

**Table 6:** Computational comparison of different infrared and visible image fusion methods on the M3FD Fusion dataset. Bold indicates the best result.

Method	Params (M)	FLOPs (G)	Inference Speed (FPS)	GPU Memory (MiB)
DenseFuse	0.0742	131.779	30.0	1025.6
U2Fusion	0.6592	1036.474	5.0	2628.6
YDTR	0.2178	491.901	4.44	1323.9
EMMA	1.5177	213.187	27.0	439.2
CAF	0.1483	232.608	55.0	966.7
LUT-Fuse	0.0734	12.055	149.3	168.6
GIFNet	0.8228	1573.180	3.5	1026.6
DAFusion	2.0180	4695.439	0.95	4457.7
Ours	0.5189	463.672	14.5	1331.3

**Table 7:** Ablation study of CRFM and EDC on the M3FD dataset. The baseline uses equal-weight feature averaging and parameter-matched convolutional replacements for the two modules. Bold indicates the best result, while underline indicates the second-best result.

Configuration	MI ↑	SD ↑	VIF ↑	$Q^{\mathrm{AB}/F}↑$	NMI ↑	${\mathrm{FMI}}_{G}↑$
Baseline	4.4225	38.6423	0.6739	0.6065	0.6379	2.2416
CRFM only	4.4548	38.7394	0.6905	0.6189	0.6421	2.3156
EDC only	4.4513	38.6586	0.6812	0.6120	0.6418	2.2734
Full model	**4.7724**	**39.0047**	**0.7526**	**0.6373**	**0.6884**	**2.6535**

**Table 8:** Ablation study of the intensity, edge, and thermal target losses on the M3FD dataset. The full loss contains all three loss terms. Bold indicates the best result, while underline indicates the second-best result. Metrics marked with ↑ are higher-is-better metrics.

Loss Configuration	MI ↑	SD ↑	VIF ↑	$Q^{\mathrm{AB}/F}↑$	NMI ↑	${\mathrm{FMI}}_{G}↑$
$L_{int}$ only	3.7551	35.4358	0.4583	0.2379	0.5450	1.1160
$L_{int}+L_{edge}$	4.5001	36.4063	0.7314	0.6371	0.6512	2.5214
$L_{int}+L_{th}$	4.0003	37.1672	0.4775	0.3283	0.5794	1.2730
Full loss	**4.7724**	**39.0047**	**0.7526**	**0.6373**	**0.6884**	**2.6535**

**Table 9:** Ablation study of the relation cues in CRFM on the M3FD Fusion test set. Bold indicates the best result. Metrics marked with ↑ are higher-is-better metrics.

CRFM Configuration	MI ↑	SD ↑	VIF ↑	$Q^{\mathrm{AB}/F}↑$	NMI ↑	${\mathrm{FMI}}_{G}↑$
Difference cue only	4.5938	38.7462	0.7284	0.6182	0.6809	2.4117
Interaction cue only	4.5147	38.8215	0.7369	0.6254	0.6835	2.5038
Both relation cues	**4.7724**	**39.0047**	**0.7526**	**0.6373**	**0.6884**	**2.6535**

**Table 10:** Ablation study of the number of fusion stages on the M3FD Fusion test set. Bold indicates the best result. Metrics marked with ↑ are higher-is-better metrics.

Number of Stages	MI ↑	SD ↑	VIF ↑	$Q^{\mathrm{AB}/F}↑$	NMI ↑	${\mathrm{FMI}}_{G}↑$
1	4.5938	38.7462	0.7284	0.6182	0.6809	2.4117
2	4.5147	38.8215	0.7369	0.6254	0.6835	2.5038
3	**4.7724**	**39.0047**	**0.7526**	**0.6373**	**0.6884**	**2.6535**
4	4.7668	38.9974	0.7512	0.6368	0.6873	2.6427
5	4.7549	38.9841	0.7487	0.6354	0.6856	2.6205

**Table 11:** Ablation study of the dynamic-kernel size on the M3FD Fusion test set. Bold indicates the best result. Metrics marked with ↑ are higher-is-better metrics.

Kernel Size	MI ↑	SD ↑	VIF ↑	$Q^{\mathrm{AB}/F}↑$	NMI ↑	${\mathrm{FMI}}_{G}↑$
3×3	**4.7724**	39.0047	**0.7526**	**0.6373**	**0.6884**	**2.6535**
5×5	4.7481	39.2865	0.7478	0.6329	0.6857	2.6179
7×7	4.7016	39.5712	0.7394	0.6258	0.6798	2.5607
9×9	4.6395	**39.8427**	0.7281	0.6159	0.6709	2.4758

## Data Availability

The M3FD, RoadScene, and TNO datasets analyzed in this study are publicly available from the original sources cited in References [17,18,19]. No new datasets were created in this study.
